# mGluR5 Is Substitutable for mGluR1 in Cerebellar Purkinje Cells for Motor Coordination, Developmental Synapse Elimination, and Motor Learning

**DOI:** 10.3390/cells11132004

**Published:** 2022-06-23

**Authors:** Maria Harbers, Harumi Nakao, Takaki Watanabe, Kyoko Matsuyama, Shoichi Tohyama, Kazuki Nakao, Yasushi Kishimoto, Masanobu Kano, Atsu Aiba

**Affiliations:** 1 Laboratory of Animal Resources, Center for Disease Biology and Integrative Medicine, Graduate School of Medicine, The University of Tokyo, 7-3-1 Hongo, Bunkyo-ku, Tokyo 113-0033, Japan; harbers-maria602@g.ecc.u-tokyo.ac.jp (M.H.); hanakao@m.u-tokyo.ac.jp (H.N.); k_nakao@iexas.med.osaka-u.ac.jp (K.N.); 2 Department of Neurophysiology, Graduate School of Medicine, The University of Tokyo, 7-3-1 Hongo, Bunkyo-ku, Tokyo 113-0033, Japan; wtakaki@m.u-tokyo.ac.jp (T.W.); kyoko_m@m.u-tokyo.ac.jp (K.M.); mkano-tky@m.u-tokyo.ac.jp (M.K.); 3 International Research Center for Neurointelligence (WPI-IRCN), The University of Tokyo Institutes for Advanced Study, The University of Tokyo, 7-3-1 Hongo, Bunkyo-ku, Tokyo 113-0033, Japan; 4 Laboratory of Physical Chemistry, Faculty of Pharma-Science, Teikyo University, 2-11-1 Kaga, Itabashi-ku, Tokyo 173-8605, Japan; 18y101321zf@stu.teikyo-u.ac.jp (S.T.); y-kishimoto@pharm.teikyo-u.ac.jp (Y.K.); 5 Institute of Experimental Animal Sciences, Graduate School of Medicine, Osaka University, 2-2 Yamadaoka, Osaka 565-0871, Japan

**Keywords:** Purkinje cells, mGluR5, mGluR1, motor coordination, synapse elimination, eyeblink conditioning

## Abstract

Group I metabotropic glutamate receptors (mGluRs) include mGluR1 and mGluR5, which are coupled to the Gq family of heterotrimeric G-proteins and readily activated by their selective agonist 3,5-dihydroxyphenilglycine (DHPG). mGluR1 and mGluR5 exhibit nearly complementary distributions spatially or temporally in the central nervous system (CNS). In adult cerebellar Purkinje cells (PCs), mGluR1 is a dominant group I mGluR and mGluR5 is undetectable. mGluR1 expression increases substantially during the first three weeks of postnatal development and remains high throughout adulthood. On the other hand, mGluR5 expression is observed during the first two postnatal weeks and then decreases. However, functional differences between mGluR1 and mGluR5 in the CNS remains to be elucidated. To address this issue, we generated “mGluR5-rescue” mice in which mGluR5 is specifically expressed in PCs in global mGluR1-knockout (KO) mice. mGluR5-rescue mice exhibited apparently normal motor coordination, developmental elimination of redundant climbing fiber (CF)-PC synapses, and delay eyeblink conditioning, which were severely impaired in mGluR1-KO mice. We concluded that mGluR5 is functionally comparable with mGluR1 in cerebellar PCs.

## 1. Introduction

Metabotropic glutamate receptors (mGluRs) are activated by neurotransmitter glutamate and coupled to heterotrimeric G-proteins. Eight subtypes (mGluR1-8) are recognized and classified into three groups, the groups I, II, and III, according to their amino acid sequences, coupled G-proteins, and pharmacological profiles [1]. Two subtypes of group I mGluRs, mGluR1 and mGluR5, are coupled to Gq-family proteins and selectively activated by an agonist 3,5-dihydroxyphenilglycine (DHPG). The activation of group I mGluRs leads to increases in phosphoinositide hydrolysis and generates diacylglycerol and inositol 1,4,5 trisphosphate (IP_3_), which induce two cellular responses: IP_3_ receptor (IP_3_R)-mediated Ca^2+^ release from the smooth endoplasmic reticulum and transient receptor potential canonical (TRPC)-mediated currents by diacylglycerol [2]. These signaling molecules play pivotal roles in the modulation and plasticity of excitatory synaptic transmission as well as neuronal development and various learnings. Despite these similarities, mGluR1 and mGluR5 exhibit nearly complementary distributions spatially or temporally. For example, mGluR1 is strongly expressed in the thalamus, hippocampal dentate gyrus, and cerebellum, while mGluR5 is mainly expressed in the cerebral cortex, striatum, and hippocampal CA1-CA3 regions [3,4]. In cerebellar PCs, mGluR1 is a dominant group I mGluRs and its protein level increases substantially during the first three weeks of postnatal development and remains high throughout adulthood [5]. In contrast, mGluR5 expression is observed during the first two weeks of postnatal development, but then it declines and becomes undetectable in adult PCs [6]. There are reports that the impaired function of mGluR1 in PCs, which cannot be replaced by mGluR5, is related to the disease. For example, the switching from mGluR1 to mGluR5 in PCs was observed in experimental autoimmune encephalomyelitis (EAE) model mice and multiple sclerosis patients as well as spinocerebellar ataxia type 1 (SCA 1) model mice. In EAE model mice, an mGluR1 positive allosteric modulator (PAM) improved motor coordination, whereas mGluR5 antagonists did not affect impaired motor coordination [7]. In addition, mGluR1 PAM caused a prolonged improvement of motor coordination, whereas mGluR5 PAM caused only a short-lasting improvement of motor coordination in SCA 1 model mice [8]. To clarify the possible functional difference between mGluR1 and mGluR5 in CNS, we took advantage of rescue experiments using mGluR1-KO mice with transgenes expressing mGluRs specifically in PCs. mGluR1-KO mice show impairments in the induction of long-term depression (LTD) of PF-PC synaptic transmission, developmental elimination of CF-PC synapses, delay eyeblink conditioning, and motor coordination [9,10]. All these defects are rescued by the selective introduction of mGluR1a receptors in PCs [11,12]. In this study, we have generated “mGluR5-rescue” mice, in which mGluR5 is expressed specifically in PCs in global mGluR1-KO mice [13]. We found that mGluR5-rescue mice restored the dysfunctions of motor coordination, CF-PC synapse elimination, and delay eyeblink conditioning in mGluR1-KO mice. Our results indicate that mGluR5 is substitutable for mGluR1 in PCs at both the synaptic and behavioral levels.

## 2. Materials and Methods

### 2.1. Animals

Animal experiments and methods were approved by and performed in accordance with the relevant guidelines and regulations of the animal welfare committees of The University of Tokyo. Animals were housed in a controlled humidity and temperature room under a 12 h light/dark cycle, with free access to food and water.

### 2.2. Generation of Transgenic Mice

To obtain rat mGluR5 cDNA fragment, we performed PCR with primer set with BamHI recognition site (5’-CGGGATCCGCCACCATGGTCCTTCTGTTGATCCTGTCAGTCCTACTTC-3’, 5’-CGGGATCCTCACAACGATGAAGAACTCTGCGTGTAATCTCTGATG-3’) using pmGR5 cDNA plasmid DNA as a template. pmGR5 (RDB13199) was provided by RIKEN BRC through the National BioResource Project of the MEXT/AMED, Japan. An L7-mGluR5 transgenic vector was constructed by inserting the rat mGluR5 cDNA fragment into a BamHI site in exon 4 of the L7 gene cassette [14,15]. The transgene construct was sandwiched between 0.23 kb + 1.2 kb and 0.23 kb insulator sequences [16]. L7-mGluR5 DNA fragments were microinjected into C57BL/6N embryos. Progeny was screened for the presence of the transgenes by PCR and Southern blot analysis of tail DNAs. PCR was performed using transgene-specific primers and endogenous mGluR1 primers (5′-GGTTTGCACAGGAGAACAGCAAG-3’, 5′-CCGTCCATCAATTGGCTTCATCGC-3′ for L7-mGluR5 and 5′-AGCTTTGAACCAGCTGTGTTGGC-3′, 5′-ATACCATACTGTTCTCTGATCTCC-3′ for mGluR1) under the following conditions: 94 °C for 2 min, 30 cycles of melting at 94 °C for 30 s, annealing at 64 °C (for L7-mGluR5) or 60 °C (for mGluR1) for 30 s, and extension at 72 °C for 30 s, with additional extension at 72 °C for 2 min at the end. PCR products were separated on 2% agarose gels. For Southern blot analysis, tail DNAs were digested with BamHI, separated on a 0.8% agarose gel and transferred to Biodyne Plus membrane (Pall Corporation, Pensacola, FL, USA). A fragment of the L7 coding region was used as the hybridization probe. The membrane was hybridized with the appropriate ^32^P-dCTP-labeled probe and hybridized bands were visualized using Bio-Imaging Analyzer (BAS-2500, Fuji Photo Film, Tokyo, Japan). L7-mGluR5 transgenic (Tg) mice were mated with mGluR1^+/−^ mice to produce mGluR1^+/−^; L7-mGluR5 Tg mice. The mGluR5-rescue (mGluR1^−/−^; L7-mGluR5 Tg) mice were generated by crossing mGluR1^+/−^ mice with mGluR1^+/−^; L7-mGluR5 Tg mice.

### 2.3. Western Blot Analysis

Cerebella were isolated and homogenized in buffer containing 0.32 M sucrose, 10 mM Tris–HCl (pH 7.4), 1 mM EDTA and protease inhibitors (cOmplete Mini, EDTA free, Roche, Mannheim, Germany). Proteins were separated by sodium dodecyl sulfate-polyacrylamide gel electrophoresis (SDS-PAGE) and transferred to Immobilon-P membrane (Millipore, Billerica, MA, USA). The membrane was incubated with anti-mGluR5 antibody (1:5000, ab76316, Abcam, Cambridge, UK) and β-actin (1:1000, AC-74, Sigma-Aldrich, St. Louis, MO, USA) followed by anti-rabbit HRP-conjugated secondary antibody (1:7000, Jackson ImmunoResearch, West Grove, PA, USA) or anti-mouse HRP-conjugated secondary antibody (1:7000, Jackson ImmunoResearch). The signals were visualized by ECL Prime detection reagents (RPN2232, Cytiva, Marlborough, MA, USA).

### 2.4. Immunohistochemical Analysis

Two- to four-months-old mice were deeply anesthetized and were perfused transcardially with 4% paraformaldehyde in 0.1 M phosphate buffer (PB pH 7.4). Brains were removed from the skull and exposed to 4% paraformaldehyde for 2 h. After washing with phosphate-buffered saline (PBS), brains were cryoprotected with 30% sucrose in 0.1 M PB and sectioned at 40 µm thickness using a freezing microtome (FX-801, Yamato, Saitama, Japan). The free-floating sections were incubated with antibodies to mGluR5 (1.0 mg/mL) [17] and calbindin (1:1000, CB300, SWANT, Burgdorf, Switzerland) overnight at room temperature. These sections were then washed with 0.5% Tween-20 in PBS (PBST) and incubated with Cy3-conjugated antibody to rabbit IgG (1:200, Jackson ImmunoResearch) and Alexa488-conjugated antibody to mouse IgG (1:400, Invitrogen, Waltham, MA, USA) for 2 h at room temperature. Photographs were taken with a fluorescence microscope (BZ-9000, Keyence, Osaka, Japan).

### 2.5. Preparation of Synaptosomal Fraction and Coimmunoprecipitation

The synaptosomal fraction from wild-type, L7-mGluR5 Tg or mGluR5-rescue cerebella was prepared as described previously [18]. The cerebellar lysates for Western blot analysis were centrifuged at 1000× *g* for 15 min, and the supernatant was centrifuged again at 10,000× *g* for 30 min. The sediment (synaptosomal fraction) was solubilized in a lysis buffer containing 50 mM Tris-HCl (pH 7.4), 150 mM NaCl, 1% NP-40, and protease inhibitor and rotated at 4 °C for 1 h, followed by centrifugation at 15,000× *g* for 40 min. For coimmunoprecipitation, the resultant supernatant of each genotype was incubated overnight with an antibody to mGluR1 (lot1 antibody) [19] or mGluR5 (AB5675, Millipore), and negative control mouse IgG or rabbit IgG. Immune complexes were then precipitated by incubation with protein G Sepharose (Amersham Pharmacia Biotech, Uppsala, Sweden) for 2 h, followed by centrifugation at 800× *g* for 1 min. Precipitated proteins were washed 2 times with lysis buffer and once with lysis buffer without a protease inhibitor. Bound proteins were immunoblotted using antibodies against mGluR5 (1:5000, ab76316, Abcam), mGluR1 (1:2500, 610965, BD Bioscience, San Jose, CA, USA), and Pan-Homer (1:1000, sc-8921, Santa Cruz, Starr County, TX, USA) followed by anti-rabbit, anti-mouse, or anti-goat (1:7000, Jackson ImmunoResearch) HRP-conjugated secondary antibodies. The signals were visualized by ECL Prime detection reagents.

### 2.6. Rotarod Test and Footprints

Two-month-old male mGluR5-rescue mice and littermate control mice (wild-type, mGluR1^+/−^ or mGluR1^+/−^; L7-mGluR5 Tg) were used for the rotarod test. Rota-Rod Treadmill (MK660C; Muromachi Kikai, Tokyo, Japan) measured motor coordination and motor learning. Mice were tested for 5 consecutive days and 3 trials per day with 15 min inter-trial intervals. Mice were placed on the rotating rod with an accelerating speed from 4 to 40 rpm at 300 s. The maximum observation time was 300 s. The latency to fall from the rod was recorded and the mean value was calculated for each day. The weight of each mouse was recorded every day after the rotarod test. A footprint test was performed last day of the rotarod test to examine the step patterns of the hind limbs during forward locomotion. The hind paws of mice were inked with dyes of black, and footprints were traced on the paper covering a runway.

### 2.7. Delay Eyeblink Conditioning

The surgical procedure for mouse eyeblink conditioning was the same as described previously [12,20,21]. Briefly, the mice were anesthetized with ketamine (80 mg/kg, i.p.; Daiichi Sankyo, Tokyo, Japan) and xylazine (20 mg/kg, i.p.; Bayer, Tokyo, Japan). Next, four Teflon-coated stainless-steel wires with a 101.6 µm coating diameter (A-M Systems, WA, USA) were surgically implanted with a left eyelid. Two of the four wires were used to acquire electromyograms (EMG) from the eyelid muscles, and the remaining two were used to deliver the unconditioned stimulus (US). Electrodes equipped with wires were mounted using dental cement (Unifast II; GC Corporation, Tokyo, Japan). Two days were allotted for recovery and acclimation to the conditioning chamber post-surgery.

Next, 2–6-month-old mGluR5-rescue mice (n = 10) and the littermate wild-type mice (n = 10) consisting of 4 male and 6 female mice were trained in the delay paradigm of eyeblink conditioning. A tone (1 kHz, 80 dB, 352 ms duration) was used as the conditioned stimulus (CS) and an electrical shock (100 Hz square pulses, 100 ms duration) as the US. The US intensity was carefully adjusted to the minimal amplitude required to evoke a constant UR. The inter-trial interval was set at a minimum of 20 s and a maximum of 40 s, with a mean value of 30 s. The data analysis methods, including the criteria for CR, startle response, and CR peak latency, were the same as previously reported [12,20,21]. The CR amplitude was defined as the EMG amplitude 50 ms before US onset. The CR onset latency was defined as the time interval from the CS onset to the time when the EMG amplitude first reached the criterion for CR. UR amplitudes were determined as the EMG amplitude 50 ms after US termination.

### 2.8. Electrophysiological Recording

Parasagittal cerebellar slices of 250 μm thickness were prepared with a vibratome slicer (VT-1200S, Leica, Wetzlar, Germany) from adult mGluR5-rescue mice. Cerebellar slices were incubated for more than 30 min at room temperature in artificial cerebrospinal fluid (ACSF) composed of 125 mM NaCl, 2.5 mM KCl, 1.25 mM NaH_2_PO_4_, 26 mM NaHCO_3_, 2 mM CaCl_2_, 1 mM MgSO_4_, and 20 mM glucose bubbled with 95% O_2_ and 5% CO_2_. Then, one of the slices was placed in a recording chamber on an upright fluorescent microscope (BX-50W, Olympus, Tokyo, Japan). The chamber was filled with oxygenated ACSF with 0.1 mM picrotoxin at 30–34 °C. Whole-cell patch-clamp recordings were made from visually identified PCs with patch pipettes (GC150T-10, Harvard Apparatus, Holliston, MA, USA) filled with an intracellular solution composed of 60 mM CsCl, 10 mM d-gluconic acid, 20 mM TEA-Cl, 30 mM 4-(2-HydroxyEthyl)-1-PiperazineEthaneSulfonic acid (HEPES), 20 mM 1,2-bis(2-Aminophenoxy)ethane-N,N,N′,N′-tetraacetic acid tetrapotassium salt (BAPTA-K4), 4 mM MgCl_2_, 4 mM Na_2_-adenosine triphosphate (ATP), and 0.4 mM Na_2_-guanosine triphosphate (GTP), which was adjusted to pH 7.3 and an osmolarity of 295 mOsm with CsOH. CFs were stimulated with another pipette filled with ACSF at several sites in the granular layer around the recorded PC. The stimulation frequency was 0.2 Hz and the stimulus pulse width was 0.1 ms. Stimulation currents were gradually increased at each site from 0 to 100 μA and evoked CF-mediated excitatory postsynaptic currents (CF-EPSCs) were recorded. The number of discrete CF-EPSC steps represents the number of CFs innervating the PC under recording.

### 2.9. Statistical Analyses

The data of the behavior tests were analyzed by repeated-measure ANOVA using GraphPad Prism 6 (GraphPad Sofware Inc., La Jolla, CA, USA) or *t*-test and expressed means ± SEM. In addition, Kruskal–Wallis test and Steel–Dwass post hoc test were used for comparing frequency distributions of the number of CF-EPSC steps from three populations of PCs. The difference was considered significant when the *p*-value was smaller than 0.05. The detailed statistics are described in Table S1.

## 3. Results

### 3.1. Generation of mGluR5-Rescue Mice

**Figure 1:**
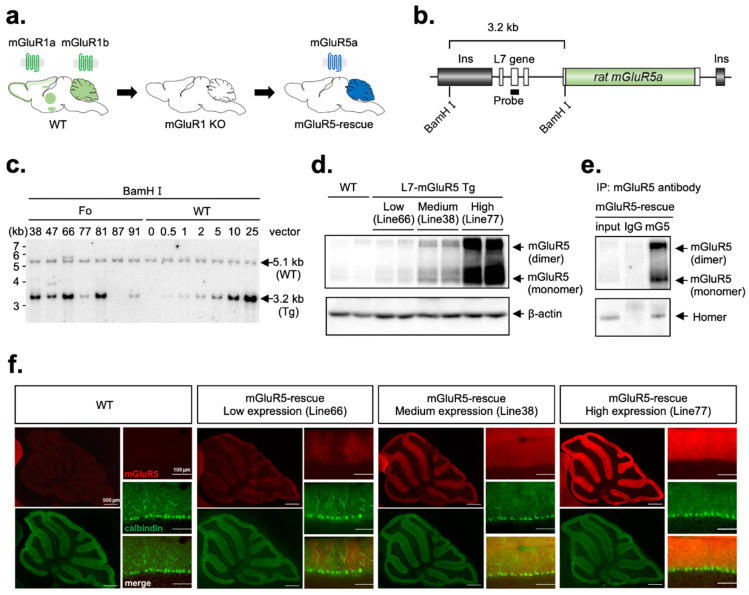
(**a**) Strategy for the generation of mGluR5-rescue mice. (**b**) Schematic structure of the transgene construct with Southern blot strategy. Rat mGluR5a cDNA was inserted into the L7 cassette. Open boxes represent exons of the L7 gene (exons 1-4). To avoid the position effect, the L7-mGluR5a transgene is flanked by insulator sequences (gray boxes) from chicken globin gene. (**c**) Southern blot analysis of tail DNA. Seven founder mice (ID: 38, 47, 66, 77, 81, 87, and 91) were obtained. We detected 5.1 kb and 3.2 kb bands derived from endogenous and transgenic L7 genes, respectively. To estimate the copy number of transgenes, the intensity of the transgene band of each transgenic mouse was compared with those of the standard DNA (wild-type (WT) mouse DNA with 0.5, 1, 2, 5, 10, and 25 copies of transgene vector DNA added per genome). (**d**) Western blot analysis of mGluR5 proteins in the cerebella of WT and L7-mGluR5 Tg mice. We obtained three transgenic lines of L7-mGluR5 Tg mice with different expression levels of mGluR5 (mGluR5 expression: Line66 < Line38 < Line77). β-actin was used as control. (**e**) Co-immunoprecipitation of mGluR5 and Homer. Synaptosomal fraction from the cerebellum of mGluR5-rescue High mice was incubated with antibody to mGluR5 (mG5). Subsequently, the input and the proteins bound to mGluR5 and IgG were immunoblotted using antibodies against the mGluR5 and Homer. (**f**) Immunohistochemical analysis for mGluR5-rescue mice. Parasagittal sections from WT and three transgenic lines of mGluR5-rescue mice (Low, Medium, and High) stained with antibody to mGluR5 (red) and antibody to calbindin (green). mGluR5 was expressed in molecular layer of cerebellar cortex in mGluR5-rescue mice.

Splice variants of mGluR5 include mGluR5a and mGluR5b, in which 32 amino acids are inserted at the C-terminus of mGluR5a. mGluR5a is a predominant splice variant in the early postnatal days in the cerebellum [22,23]. We first introduced a transgene (L7-mGluR5a) that expressed mGluR5a under the control of the PC-specific L7 promoter into the wild-type mice (Figure 1a,b). Seven founders of the L7-mGluR5a Tg mouse were obtained (Figure 1c). Western blot analysis showed that three lines of L7-mGluR5a Tg mice, Line 66, 38, and 77, expressed mGluR5 protein in the cerebella (Figure 1d). We confirmed that mGluR5 was restricted to the cerebellar PCs in L7-mGluR5a Tg mice (Figure 1f and Figure S1). Three Tg lines expressing various amounts of mGluR5 are referred to as Low, Medium, and High L7-mGluR5a Tg mice (Figure 1d,f). Next, we crossed these Tg mice with mGluR1-KO mice to obtain mGluR5-rescue mice in which mGluR5 is expressed specifically in PCs in the mGluR1-KO background (mGluR1^−/−^; L7-mGluR5 Tg). We compared the expression of High L7-mGluR5a transgene mRNA in the cerebellum of mGluR5-rescue mice and that of mGluR1 mRNA in the wild-type cerebellum using quantitative real-time PCR analysis of reverse transcripts of these mRNAs (Figure S2). We found that amount of mGluR5 mRNA in mGluR5-rescue PCs is comparable to that of mGluR1 mRNA in the control PCs. A scaffolding protein Homer binds to the C-terminal domain of long splice variants of group I mGluRs, mGluR1a, mGluR5a, and mGluR5b and regulates the localization and function of these receptors [24]. To examine whether mGluR5 expressed ectopically in mGluR1-deficient PCs binds to Homer proteins as mGluR1 does, we performed immunoprecipitation. We confirmed that Homer proteins were included in the mGluR5 protein complexes in the mGluR5-rescue cerebellar lysates (Figure 1e). We then compared the binding of Homer to mGluR1a and/or mGluR5a in the different mGluR1 backgrounds: the cerebella lysates from wild-type (mGluR1^+/+^; non Tg), wild-type with L7-mGluR5a Tg (mGluR1^+/+^; Tg), and mGluR5-rescue (mGluR1^−/−^; Tg) mice (Figure S3). In mGluR1 protein complexes precipitated with mGluR1 antibody in mGluR1^+/+^ and mGluR1^+/+^; Tg mice, we found no significant difference in the amounts of Homer between two genotypes (Figure S3a). mGluR1 and mGluR5 are co-precipitated by mGluR1 or mGluR5 antibody, suggesting that these receptors form a heterodimer (R1/R5) in mGluR1^+/+^; Tg cerebella, as previously observed in vivo and transfected cultured cells (Figure S3a,b) [25,26]. In contrast, it was observed that the amounts of Homer in mGluR5 protein complexes of mGluR1^−/−^; Tg mice precipitated with mGluR5 antibody were significantly less than that in mGluR1^+/+^; Tg mice that contained mGluR1/5 heterodimers in spite of similar amounts of mGluR5 in mGluR5 complexes from both genotypes (Figure S3b). These results suggested that mGluR5 homodimers may bind less efficiently to Homer than mGluR1/5 heterodimer or mGluR1 homodimer complexes in PCs.

### 3.2. Normal Motor Coordination in mGluR5-Rescue Mice

**Figure 2:**
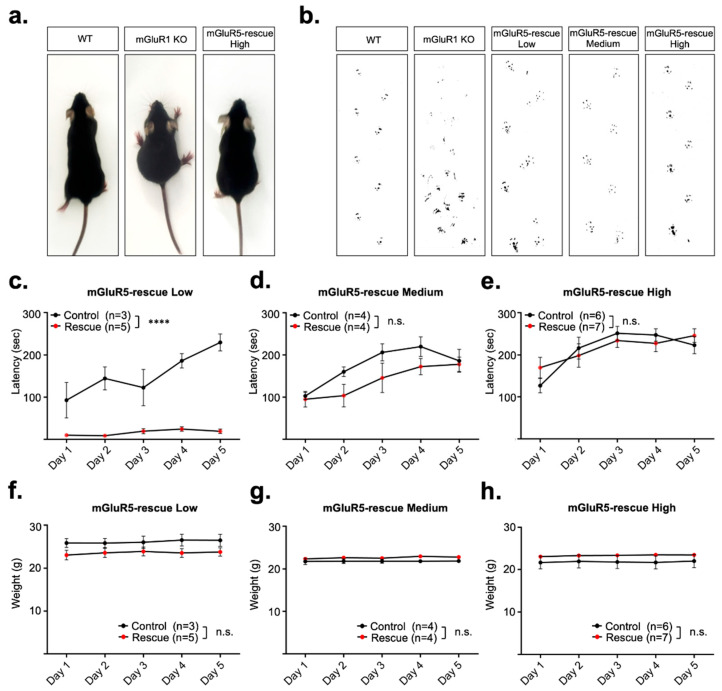
(**a**) Photographs of a wild-type (WT), an mGluR1-KO and an mGluR5-rescue High mouse are shown. mGluR1-KO mouse shows hindlimb spreading, which is improved in mGluR5-rescue mouse. (**b**) Hind limb footprint patterns. (**c**–**e**) Rotarod test in control mice and mGluR5-rescue Low (**c**), Medium (**d**), and High (**e**) mice. The latency to fall from a rotating rod is plotted versus the training day. (**f**–**h**) Weight of mice used in the rotarod test on each test day is shown. Low (**f**), Medium (**g**) and High (**h**). Data are expressed as mean ± SEM. **** *p* < 0.0001. n.s., not significant.

The mGluR1-KO mice show severe ataxia, suggesting that mGluR1 is required for motor coordination [9]. We performed the rotarod test and footprint analysis to determine whether the impaired motor coordination observed in the mGluR1-KO mice is rescued in mGluR5-rescue mice. mGluR5-rescue mice exhibited apparently normal step patterns as mGluR5 expression increased, although mGluR1-KO mice showed hindlimb spreading (Figure 2a,b). In the rotating rod task, mGluR5-rescue mice learned how to keep themselves on the rod depending on the amount of mGluR5 protein (Figure 2c–e). There was no significant difference in body weight between control and mGluR5-rescue mice (Figure 2f–h). These results suggest that mGluR1 in PCs is functionally replaceable by mGluR5 for motor coordination.

### 3.3. Normal Regression of Multiple CF Innervation in PCs of mGluR5-Rescue Mice

**Figure 3:**
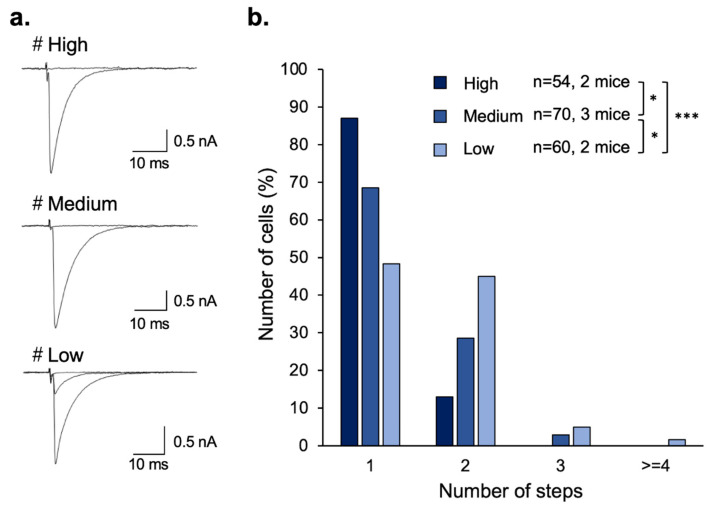
Sample records (**a**) and summary graphs (**b**) of CF-EPSCs from adult mGluR5-rescue PCs. Summary graphs show frequency distributions of PCs in terms of the number of discrete steps of CF-EPSCs from mGluR5-rescue High (n = 54 from 2 mice), Medium (n = 70 from 3 mice), and Low (n = 60 from 2 mice) mice. * *p* < 0.05, *** *p* < 0.005.

Persistent multiple CF innervation of PCs in mGluR1-KO mice suggests that mGluR1 is essential for the developmental transition from multi- to mono-innervation of PCs by CFs [10]. We investigated whether mGluR5 signaling was substitutable for mGluR1-dependent CF synapse elimination. We made whole-cell recordings from PCs, stimulated CFs and recorded CF-EPSCs in cerebellar slices from adult mice. We estimated the number of CFs innervating each PC from discrete CF-EPSCs elicited in each PC. In mGluR5-rescue High mice, more than 80% of PCs were innervated by single CFs, which is almost the same percentage as the wild-type mice (Figure 3a,b) [27]. As well as motor coordination, multiple CF innervation of PCs in mGluR1-KO mice was rescued differently depending on the amount of mGluR5 protein (Figure 3a,b). These results indicate that mGluR5 can drive the signaling cascades responsible for CF synapse elimination in the absence of mGluR1.

### 3.4. Recovered Performance of Eyeblink Conditioning as a Motor Learning in mGluR5-Rescue Mice

**Figure 4:**
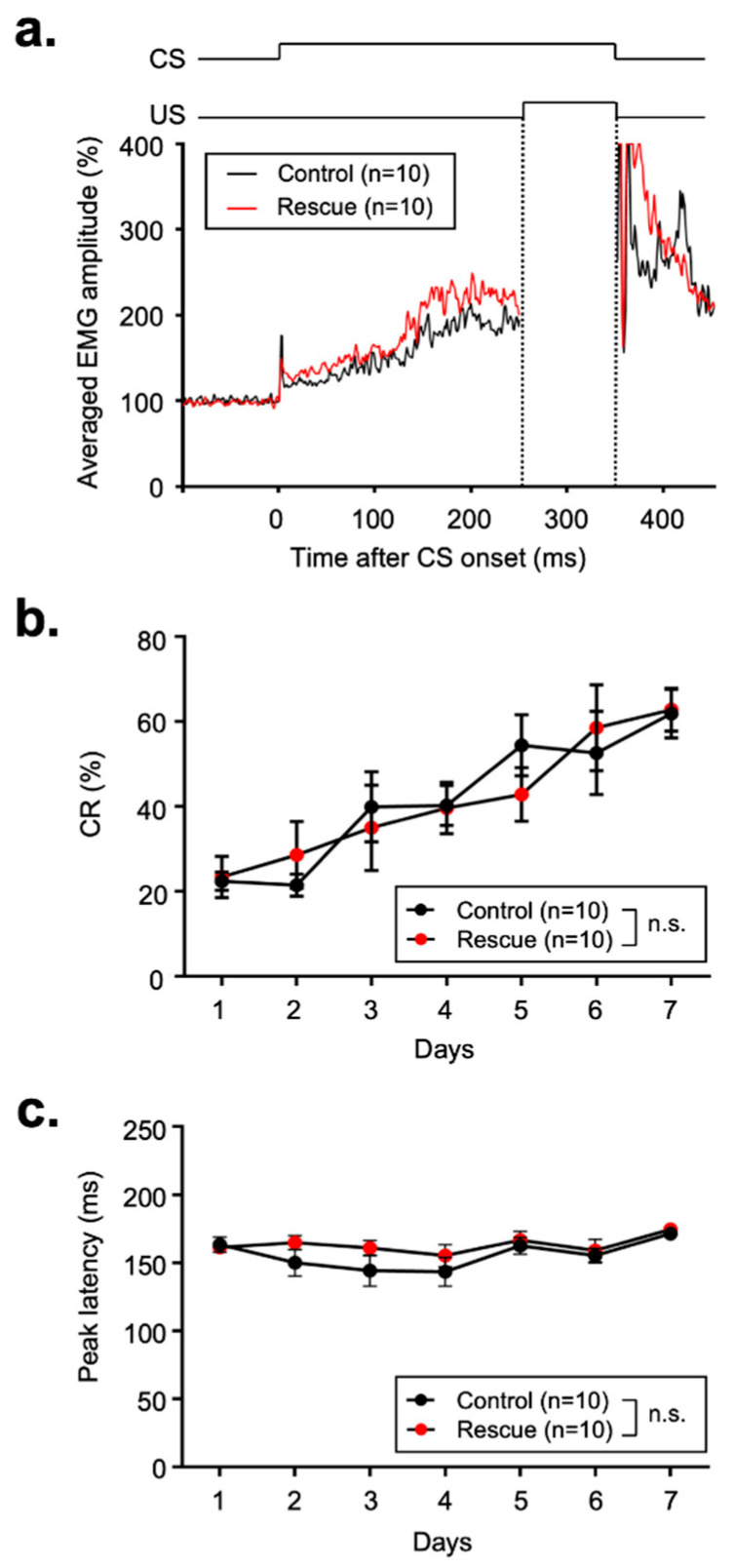
(**a**) Temporal relationship between the CS and US in eyeblink conditioning. A temporal overlap of the US with the preceding CS can be observed. The averaged EMGs of control mice and mGluR5-rescue mice were imposed under the CS-US time course. (**b**) CR acquisition as a motor learning curve in the control mice and mGluR5-rescue mice. In the acquisition session of day 1 to day 7, daily session consisted of 10 blocks of trials, with each block consisting of nine CS–US paired trials. (**c**) CR peak latency during the eyeblink conditioning in the control and mGluR5-rescue mice. In this analysis, we used mGluR5-rescue High mice. Data are expressed as mean ± SEM. n.s., not significant.

mGluR1-KO mice show a deficit in delay eyeblink conditioning, and the deficit is restored by the introduction of mGluR1a into cerebellar PCs. This demonstrates that the mGluR1 within the cerebellar PCs is essential for conditioned response (CR) acquisition in delay eyeblink conditioning [12,18,21]. We tested delay eyeblink conditioning in the control and mGluR5-rescue High mice to investigate whether mGluR5 could substitute the function of mGluR1 in delay eyeblink conditioning (Figure 4). Classical conditioning lasted for 7 days, and the magnitude of the CR on the last day did not differ between the control and mGluR5-rescue mice (Figure 4a). In the previous studies, the CR% on the last day was less than 40% in mGluR1-KO mice, whereas CR% for both control and mGluR5-rescue mice progressively increased during the 7-day acquisition session and reached over 60% on the last day (Figure 4b) [12,18]. ANOVA revealed no significant interaction effects between sessions and genotypes, and there were no significant genotypic effects. Post hoc analysis also revealed no significant differences between the control and mGluR5-rescue mice in any session. Next, we analyzed CR peak latencies during the 7-day training session (Figure 4c). ANOVA revealed no significant interaction effects between sessions and genotypes, and there were no significant genotypic effects, although there was a tendency for longer latency in the early stage of the acquisition session. Other indices, such as CR amplitude, CR onset latency, UR amplitude, and startle reflex frequency, were not significantly different between the two groups (Table 1). Of these indices, the averaged CR amplitude of mGluR5-rescue mice was greater than that of control mice (248.98 ± 41.07 vs. 204.81 ± 30.1, *p* = 0.4, *t*-test). However, we do not consider it necessary to focus on this difference, because CR amplitude is highly variable and there was no significant difference between two genotypes. Taking these results together, we conclude that the impairment of eyeblink conditioning previously observed in mGluR1-KO mice was restored by the PC-specific expression of mGluR5.

The Comparison of eyeblink conditioning parameters. The CR amplitude, CR onset latency, UR amplitude, and startle response were calculated on day 7 in the control and mGluR5-rescue mice. Values are group mean ± SEM.

## 4. Discussion

In this study, we addressed the question of whether mGluR1 and mGluR5 have distinct functions in vivo, especially in cerebellar PCs. We generated mGluR5-rescue mice and demonstrated that mGluR5 was substitutable for mGluR1 in PCs for motor coordination, developmental synapse elimination, and motor learning. The ectopic expression of mGluR5a in PCs restored the abnormalities of CF synapse elimination, motor learning, and motor coordination in mGluR1-KO mice, phenocopying mGluR1a-rescue mice [11,12,18]. Although both mGluR1 and mGluR5 are Gq-coupled receptors that stimulate intracellular Ca^2+^ release, they differ in the kinetics of intracellular Ca^2+^ release in response to receptor activation in ectopically expressed cultured cells. It has been shown that activation of mGluR1 evokes single-peaked Ca^2+^ mobilization, while mGluR5 activation leads to Ca^2+^ oscillations [28]. In this study, however, mGluR5 could drive the signaling pathway that can substitute the functions of mGluR1-signaling in PCs.

Alternative splicing generates two variants of mGluR5: mGluR5a and mGluR5b. mGluR5a is the predominant isoform in the early postnatal days in the cerebellum [22,23], whereas mGluR5b is the predominant isoform in most brain regions in adulthood [22,23,29]. In this study, we expressed mGluR5a in PCs from early postnatal to adult stages. It would be of interest to see whether mGluR5b does the same as mGluR5a. Although they have a similar pharmacological profile, the formation and maturation of neurites differ in NG 108-15 cells. mGluR5a hinders the acquisition of mature neuronal traits, and mGluR5b fosters the elaboration and extension of neurites [30]. For further understanding of the functions of mGluR5 splice variants in vivo, it would be interesting to generate mGluR5b-rescue mice where mGluR5b is expressed specifically in PCs in the mGluR1-KO background and examine the rescue efficacy of mGluR5b.

There are reports which showed the change in the expression from mGluR1 to mGluR5 in PCs of disease model mice with cerebellar dysfunction. For example, mice developing EAE showed impairment in motor coordination and a progressive reduction in mGluR1 expression in PCs, associated with increased mGluR5 expression in PCs. Such switching from mGluR1 to mGluR5 in PCs was also observed in multiple sclerosis patients and SCA 1 model mice. In the EAE model mice, mGluR1 PAM improved motor coordination, whereas mGluR5 antagonists did not affect impaired motor coordination [7]. SCA 1 model (SCA1154Q/2Q) mice showed a progressive degeneration of PCs, impaired motor coordination, and premature death. mGluR1 PAM caused a prolonged improvement of motor coordination, whereas mGluR5 PAM caused only a short-lasting improvement of motor coordination in the SCA 1 model mice [8]. These results suggested possible functional differences in mGluR1 and mGluR5 signaling in the disease model mice. We should consider some points to discuss the functional differences based on the results in these model mice. The responsiveness of mGluR1- and mGluR5-specific drugs to the mGluR1–mGluR5 heterocomplex needs to be confirmed because of the co-expression of mGluR1 and mGluR5 in the PCs of these mice. Quantifying the expression of mGluR1 and mGluR5 in PCs is also necessary for precise discussion. The three L7-mGluR5 transgenic mouse lines in this study and mGluR1-KO mice allow us to generate mice with PCs that express varying amounts of mGluR1 and mGluR5 (Figure 1d,f and Figure S3). Future experiments using these mice might resolve these points.

We confirmed that Homer proteins were included in mGluR5a protein complexes prepared from mGluR5-rescue cerebellar lysates, suggesting that ectopically expressed mGluR5 can generate a signaling complex that is similar to endogenous mGluR1 in PCs. Furthermore, our biochemical analysis showed that mGluR5 homodimers might bind less efficiently to Homer than mGluR1/5 heterodimer or mGluR1 homodimer complexes in PCs. We previously generated mGluR1a-P/E-rescue mice expressing mGluR1a with a point mutation (proline (P) 1153 to glutamate (E)) in its Homer binding domain. We found that the P/E-mutant mGluR1a deficient for Homer binding did not significantly alter its distribution at perisynaptic sites of parallel fiber-PC synapses and rescued impaired motor coordination observed in mGluR1-KO mice [31]. Taken together with the lower amount of Homer binding with mGluR5 homodimers in mGluR5-rescue mice, the binding of Homer to mGluR1/5 may not contribute significantly to group I mGluR function in PCs.

Several points should be clarified by future studies. First, mGluR1-mediated synaptic responses and plasticity at parallel fiber-PC synapses were not examined in this study. The electrophysiological analyses of IP_3_R-mediated Ca^2+^ release, TRPC3-mediated inward currents, endocannabinoid-mediated retrograde synaptic suppression, and LTD induction in mGluR5-rescue mice would show functional redundancy between mGluR1 and mGluR5 more clearly. Secondly, we have not examined the dose-dependency of the eyeblink conditioning test. We found that phenotypes of motor coordination and synapse elimination depended on the expression levels of mGluR5 in PCs. In this study, we found that the eyeblink conditioning was restored in mGluR5-rescue High mice. It would be interesting to examine whether the eyeblink conditioning also depends on the amount of mGluR5 in PCs by using the other two L7-mGluR5 lines: Low and Medium. Thirdly, functional redundancy between mGluR5 and mGluR1 was examined only in cerebellar PCs in this study. We previously showed that trace eyeblink conditionings, in which interstimulus intervals between CS and US were 250 or 500 ms, were impaired in mGluR1-KO and mGluR1a-rescue mice [12]. We also examined the social transmission of food-preference and novel-object-recognition memory tests and found that these tasks required mGluR1 in brain regions other than cerebellar PCs. Rescue experiments of these PC-independent mGluR1-KO phenotypes by ectopic expression of mGluR5 would clarify whether there is functional redundancy between mGluR5 and mGluR1 in other brain regions.

## 5. Conclusions

This study demonstrates that mGluR5 is substitutable for mGluR1 in PCs for motor coordination, developmental synapse elimination. and eyeblink conditioning. Therefore, despite several reports suggesting possible functional differences between mGluR1 and mGluR5, their function may be redundant in vivo.

## Figures and Tables

**Table 1:** The comparison of eyeblink conditioning parameters between control and mGluR5-rescue mice.

	Control		mGluR5-Rescue		
	Average	SEM	Average	SEM	*t*-Test vs. Control
CR amplitude (%)	204.81	30.1	248.98	41.07	0.4
CR onset latency (ms)	48.05	2.3	48.13	3.06	0.98
UR amplitude (%)	263.5	58.52	287.75	60.75	0.78
startle response (%)	6.05	2.35	5.17	3.08	0.82

## Data Availability

The data presented in this study are available upon request from the corresponding author.

## Supplementary Materials

The following supporting information can be downloaded at: https://www.mdpi.com/article/10.3390/cells11132004/s1, Figure S1: Immunohistochemical analysis for mGluR5-rescue mice; Figure S2: RT-qPCR analysis of cDNA from mGluR1 and mGluR5 mRNAs; Figure S3: Co-immunoprecipitation of mGluR5, mGluR1, and Homer. Table S1: Statistical analysis related to Figure 2, Figure 3, Figure 4 and Figure S2.
