# Supplementary material for: mGluR5 Is Substitutable for mGluR1 in Cerebellar Purkinje Cells for Motor Coordination, Developmental Synapse Elimination, and Motor Learning
Source: Cells. 2022 Jun 23;11(13):2004. doi: 10.3390/cells11132004 (PMC9265771; doi:10.3390/cells11132004)
Supplement: Supplementary file 1 [file cells-11-02004-s001.zip › cells-1757124-supplementary.pdf]

cells

MDPI

mGluR5 is substitutable for mGluR1 in cerebellar Purkinje cells for motor coordination, developmental synapse elimination, and motor learning

Maria Harbers [1], Harumi Nakao [1], Takaki Watanabe [2,3], Kyoko Matsuyama [2], Shoichi Tohyama [4], Kazuki Nakao [1,5], Yasushi Kishimoto [4], Masanobu Kano [2,3] and Atsu Aiba [1]

1. Laboratory of Animal Resources, Center for Disease Biology and Integrative Medicine, Graduate School of Medicine, The University of Tokyo, 7-3-1 Hongo, Bunkyo-ku, Tokyo, 113-0033, Japan; aiba@m.u-tokyo.ac.jp
2. Department of Neurophysiology, Graduate School of Medicine, The University of Tokyo, 7-3-1 Hongo, Bunkyo-ku, Tokyo, 113-0033, Japan
3. International Research Center for Neurointelligence (WPI-IRCN), The University of Tokyo Institutes for Advanced Study, The University of Tokyo, 7-3-1 Hongo, Bunkyo-ku, Tokyo, 113-0033, Japan
4. Laboratory of Physical Chemistry, Faculty of Pharma-Science, Teikyo University, 2-11-1 Kaga, Itabashi-ku, Tokyo 173-8605, Japan
5. Institute of Experimental Animal Sciences, Graduate School of Medicine, Osaka University, 2-2 Yamadaoka, Suita, Osaka, 565-0871, Japan

* Correspondence: aiba@m.u-tokyo.ac.jp

Supplemental Methods

Real-time (RT) quantitative PCR (qPCR) analysis of reverse transcripts

Total RNA was extracted from each mouse cerebellum using TRIzol reagent (Thermo Fisher Scientific, Cleveland, OH, USA). After treating with deoxyribonuclease I to digest contaminating genomic DNA, 0.5 µg total RNA was reverse transcribed using an RNA PCR Kit (AMV) ver 3.0 (Takara Bio, Shiga, Japan) according to the manufacturer's instruction. Serial dilution of quantified plasmid-DNA carrying rat mGluR1 cDNA or rat mGluR5 cDNA was used to generate the absolute standard curve for RT-qPCR. RT–qPCR analysis was performed using Go Taq 2-step RT–PCR system (Promega, Madison, WI, USA) in a Step One Plus (Applied Biosystems, Waltham, MO, USA). Primers for mGluR1 were designed to amplify both mouse and rat mGluR1 cDNA. Primers for mGluR5 were designed to amplify cDNA from L7-mGluR5 transgene mRNA, but not that from mouse mGluR5 mRNA. The primer sequences were as follows: 5′-AACACCTTCCTCAACATTTTCCGG-3′ and 5′-TTGGTCTTCACGTGCACAGAGAG-3′ for mGluR1, 5′-ACGACCTGACAGGCTGTGAC-3′ and 5′-TGCTGGAGGACTTGACCACC-3′ for mGluR5. The thermal cycling profile consisted of three stages: 94℃ for 30 s, 60℃ for 30 s, and 72℃ for 30 s, which were repeated for 40 cycles. The data of the RT-qPCR was analyzed by student *t*-test.

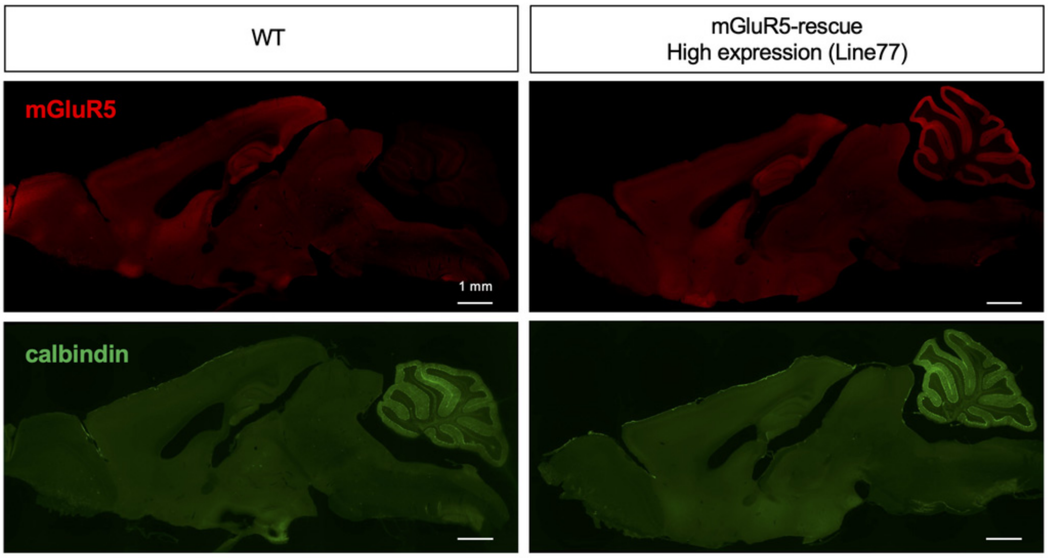

Figure S1. Immunohistochemical analysis for mGluR5-rescue mice. Whole-brain parasagittal sections from wild-type (WT) and mGluR5-rescue High mice stained with antibody to mGluR5 (red), and antibody to calbindin (green). Ectopic expression of mGluR5 by the transgene was observed specifically in the cerebellar cortex of mGluR5-rescue mice (top right).

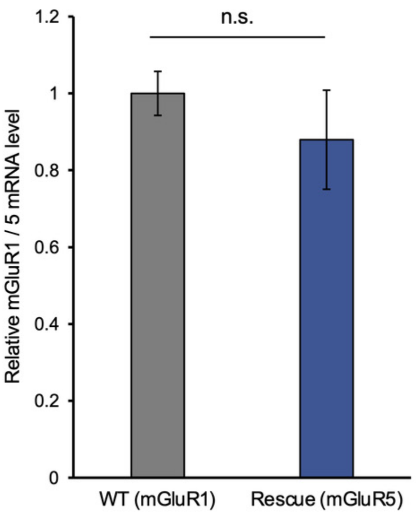

Figure S2. RT-qPCR analysis of cDNA from mGluR1 and mGluR5 mRNAs. We carried out RT-qPCR analysis of cDNA from mGluR1 mRNA in the cerebellum of wild-type (WT) mice (n = 3) and cDNA from L7-mGluR5 mRNA in the cerebellum of mGluR5-rescue High (Rescue) mice (n = 3). Standard curves were generated from serially diluted plasmid DNA and used to estimate the expression levels. Relative expression levels were shown as the ratio of mGluR5 in Rescue versus mGluR1 in WT. Data are expressed as mean ± SEM. n.s., not significant.

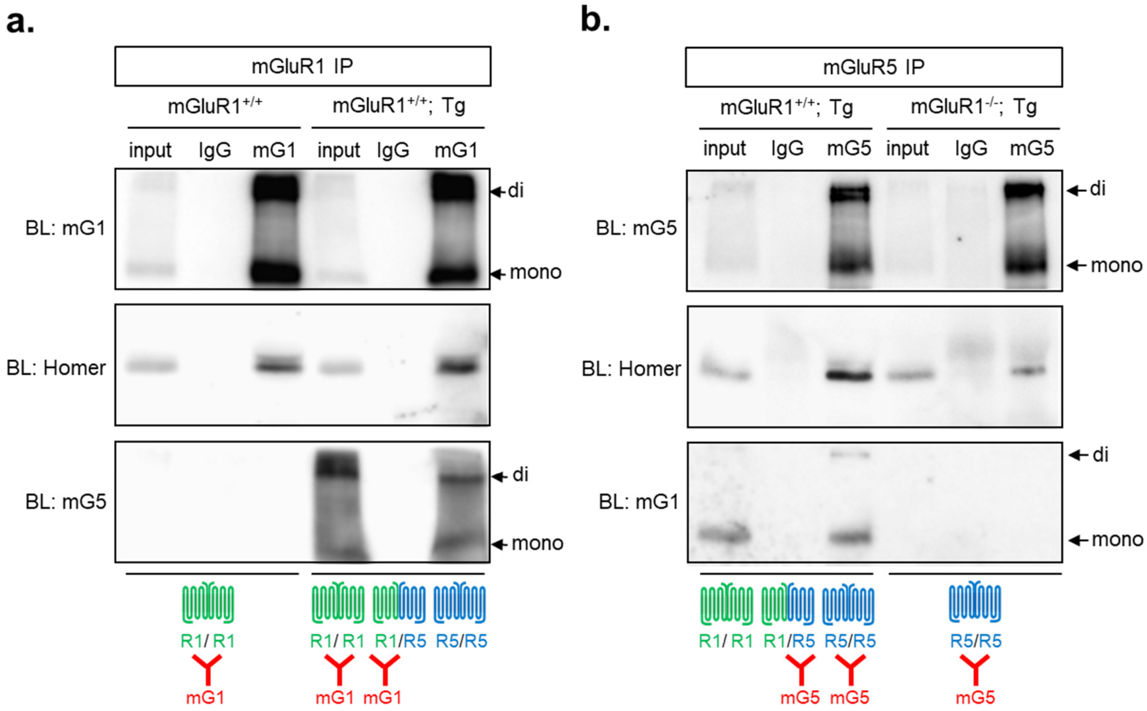

Figure S3. Co-immunoprecipitation of mGluR5, mGluR1 and Homer. A synaptosomal fraction from the cerebellum of each genotype was incubated with anti-mGluR1 (mG1) (**a**) or anti-mGluR5 (mG5) (**b**) antibodies. For this analysis, we used L7-mGluR5 Tg mice with high levels of mGluR5 expression. Subsequently, the input and the proteins bound to mGluR1 or mGluR5 antibody and IgG were immunoblotted (BL) using antibodies against the mGluR5, mGluR1 and Homer. Antibodies to the mGluR1 and mGluR5 detected both the monomers (mono) and dimers (di).

Table S1. Statistical analysis related to Figures 2-4, and Figure S2.

Figure 2c	Two-way repeated measures ANOVA.<br>Genotype: $F_{1,6}$ = 85.39, *****p* = 0.00009070;<br>control: n = 3, rescue: n = 5.
Figure 2d	Two-way repeated measures ANOVA.<br>Genotype: $F_{1,6}$ = 2.469, *p* = 0.1671;<br>control: n = 4, rescue: n = 4.
Figure 2e	Two-way repeated measures ANOVA.<br>Genotype: $F_{1,11}$ = 0.0123, *p* = 0.9137;<br>control: n = 6, rescue: n = 7.
Figure 2f	Two-way repeated measures ANOVA.<br>Genotype: $F_{1,6}$ = 2.412, *p* = 0.1714;<br>control: n = 3, rescue: n = 5.
Figure 2g	Two-way repeated measures ANOVA.<br>Genotype: $F_{1,6}$ = 2.066, *p* = 0.2007;<br>control: n = 4, rescue: n = 4.
Figure 2h	Two-way repeated measures ANOVA.<br>Genotype: $F_{1,11}$ = 1.102, *p* = 0.3163;<br>control: n = 6, rescue: n = 7.
Figure 3b	Kruskal-Wallis test. *****p* = 0.0000453;<br>Steel-Dwass post hoc test.<br>High vs Medium, **p* = 0.03886<br>High vs Low, *****p* = 0.00002932<br>Medium vs Low, **p* = 0.04612<br>High: n = 54 from 2 mice, Medium: n = 70 from 3 mice, Low: n = 60 from 2 mice.
Figure 4b	Two-way repeated measures ANOVA.<br>Genotype × session: $F_{6,108}$ = 0.462, *p* = 0.835<br>Genotype: $F_{1,18}$ = 0.00469, *p* = 0.946<br>control: n = 10, rescue: n = 10.
Figure 4c	Two-way repeated measures ANOVA.<br>Genotype × session: $F_{6,108}$ = 0.553, *p*= 0.825<br>Genotype: $F_{1,18}$ = 3.999, *p* = 0.0608<br>control: n = 10, rescue: n = 10.
Figure S2	Student *t*-test. *p* = 0.513.<br>WT: n = 3, rescue: n = 3.
